# Where You Are Is Who You Are? The Geographical Account of Psychological Phenomena

**DOI:** 10.3389/fpsyg.2020.00536

**Published:** 2020-03-24

**Authors:** Hao Chen, Kaisheng Lai, Lingnan He, Rongjun Yu

**Affiliations:** ^1^Department of Social Psychology, Zhou Enlai School of Government, Nankai University, Tianjin, China; ^2^School of Journalism and Communication, Jinan University, Guangzhou, China; ^3^School of Communication and Design, Sun Yat-sen University, Guangzhou, China; ^4^Department of Psychology, National University of Singapore, Singapore, Singapore

**Keywords:** geographical psychology, personality, cultural values, big data, well-being

## Abstract

Geographical psychology aims to study the spatial distribution of psychological phenomenon at different levels of geographical analysis and their relations to macro-level important societal outcomes. The geographical perspective provides a new way of understanding interactions between humankind psychological processes and distal macro-environments. Studies have identified the spatial organizations of a wide range of psychological constructs, including (but not limited among) personality, individualism/collectivism, cultural tightness-looseness, and well-being; these variations have been plotted over a range of geographical units (e.g., neighborhoods, cities, states, and countries) and have been linked to a broad array of political, economic, social, public health, and other social consequences. Future research should employ multi-level analysis, taking advantage of more deliberated causality test methods and big data techniques, to further examine the emerging and evolving mechanisms of geographical differences in psychological phenomena.

## Introduction

Lives are lived out in neighborhoods, cities, and states, and the physical and social features of these places can affect the behaviors, thoughts, and emotions experienced (Rentfrow, 2013). Over the past ten years, there has been a resurgence of work looking at the links between people’s psychological characteristics and the features of the places in which they live. This reinvigorated perspective and field at its third wave for history and now, now named and known as geographical psychology, aims to understand psychological phenomena based on their spatial distribution and their interactions with macro-level features of environments (Rentfrow, 2013; Rentfrow and Jokela, 2016). Its (latest) recurrence has been nurtured together by several parallels but related branches emerging in psychology in the past decade, including within-nation research in geographic clustering of personality characteristics (Rentfrow et al., 2008; Rentfrow, 2010; Rentfrow and Jokela, 2016), the trend investigating the socio-ecological causes of cultures (Fincher et al., 2008; Van de Vliert, 2009; Sng et al., 2018), big data research in spatial organizations of psychological constructs proxied by social media or online query data (Mitchell et al., 2013; Eichstaedt et al., 2015; Wu et al., 2018). The aim of this review is to overhaul how geographical psychology paves a new way of understanding human behavior through geographic and aggregate perspectives to implement this area of research at the macro level. Merits and caveats of using a geographical account to understand psychological phenomena are discussed.

## Three Waves of Recurring Interests in Geographical Perspective in Psychology

During the 1940s to 1960s, anthropological research and psychoanalytic views on personality influenced and shaped research interested in looking at psychological characteristics across nations (Rentfrow et al., 2008). However, numerous research studies (Buchanan and Cantril, 1953; McClelland, 1961) were criticized because they were lack of and not supported by theoretical explanations of how national differences emerge, persist, and are expressed at the geographical level (Inkeles and Levinson, 1969; Rentfrow et al., 2008). Thereby the first wave of geographical research in psychology has receded for several decades.

The development of personality theories and tools [e.g., establishment of the Five Factor Model (FFM)] resulted in a recurrence of interest in looking at national differences in psychological characteristics in the 1990s and 2000s. In particular, the FFM has strong biological support (Jang et al., 1998; Funder, 2001) and has been identified in several cultures (McCrae and Costa, 1997; McCrae et al., 1998), thereby providing a means for assessing and comparing national differences in personality.

Additionally, at the same time, significant evidence for psychological differences in cross-cultural research was emerging (Benet-Martínez and Oishi, 2008), thereby drawing attention to this area of research (Barenbaum and Winter, 2008). For example, Hofstede compared four important cultural dimensions – individualism and collectivism, masculinity and femininity, power distance, and uncertainty avoidance – across 50 countries and three regions (Hofstede, 2001). Research on cultural differences across nations has shown that geographical clustering can have a significant impact on the development of psychological processes (Smith et al., 2006). Moreover, geographical perspective addresses the role of socio-ecological environments, both antecedents and consequences of foci variables, has been neglected by traditional psychology research. Incorporating geography back into psychology research resulted in uncovering many crucial correlatives of personality traits (Schmitt et al., 2007), cultural values (Inglehart and Baker, 2000; Schwartz, 2008) and subjective well-being (Diener et al., 1995) across nations. Therefore, personality and cross-cultural research across nations together had brought about the second wave of recurring interest in geographic perspective.

Beyond research on cross-national differences, new work is also emerging to looking at regional psychological differences within nations. For example, Vandello and Cohen (1999) found that individualism and collectivism vary across regions within the United States. Studies that conduct research across regions and states within nations, are also referred to as within-culture studies or regional studies (Su and Ren, 2014). Regional studies in the United States have inspired research across regions and states in other countries. For example, collectivism was measured across 47 prefectures in Japan (Yamawaki, 2012) and across 15 provinces in China (Van de Vliert et al., 2013). Additionally, theoretical support from personality psychology and cross-cultural research, and previously empirical research (e.g., Krug and Kulhavy, 1973), have together inspired recent studies looking at personality differences across nine multistate regions in the United States (Plaut et al., 2002) and across U.S. states using online survey data (Rentfrow et al., 2008). In addition to research in the United States, personality differences have been examined across regions in the United Kingdom using 40 million people’s Big Five personality data via BBC online test platform (Rentfrow et al., 2015). In comparison to cross-cultural research, controlling of confounders (e.g., historical, religious, ethnic) and data collections of corresponding indicators of antecedents and outcomes are more easily handled for within-cultural research (Rentfrow et al., 2008; Rentfrow, 2010).

Almost at the same period, within-nation geographical research based on large-scale online data was emerging, and some scholars pioneered to employ digital traces on social media (i.e., Twitter and Facebook) or search engine (i.e., Google and Yahoo) by millions of users, to proxy psychological and behavioral variables (e.g., emotions, happiness, and status seeking, etc.), and showed the regional distributions across U.S. states, counties, or cities of these proxies are associated with many important societal consequences, like heart disease mortality, education, economic inequity, and so on (Mitchell et al., 2013; Eichstaedt et al., 2015; Wu et al., 2018).

Research has demonstrated uneven geographical distributions in several important factors, such as mental health, happiness, attitudes, and identity, which issues that lie at the heart of psychological science. As the accumulation of studies looking at the spatial distribution of psychological phenomena at different levels of geographical analysis, Rentfrow and his colleagues have proposed the term *Geographical Psychology* to highlight the influence of geographical perspective in understanding how psychological processes interact with macro environmental characteristics (Rentfrow, 2013; Rentfrow et al., 2015; Rentfrow and Jokela, 2016). More broadly, it proclaimed the third wave of recurring interest in geographical perspective has already arrived in psychological science.

## The Causes of Geographical Differences in Psychological Phenomena

We would present the probable causes of geographical differences in psychological phenomena in this section summarized firstly by Rentfrow et al. (2008) and Rentfrow and Jokela (2016). The causes and the processes by which psychological characteristics become spatially clustered have been explained in terms of three main mechanisms: (1) selective migration, (2) ecological influence, and (3) social influence. Selective migration mechanisms look at how individual psychological characteristics influence the environment people select. Ecological influence and social influence look at how external forces affect psychological processes and developments.

According to selective migration, people immigrate to satisfy and reinforce their basic psychological needs (Rentfrow, 2010). Hence, despite having new residents who came from places with different personalities, the geographical distribution of personality remains consistent because of genetic drift and reinforcement by the personalities of immigrants who identify themselves with their place of residence (Hofstede and McCrae, 2004; Rentfrow et al., 2015).

Ecological influence looks at how features of natural and built environments can affect human psychological processes and behaviors (Oishi and Graham, 2010; Oishi, 2014; Rentfrow and Jokela, 2016). For example, extraversion and happiness were found to be inversely correlated with the number of mountains present in the state (Oishi et al., 2015). The ecological perspective is important in explaining causes of psychological differences. For example, the pathogen-stress theory has been used to explain the causes of differences in individualism-collectivism. The pathogen theory suggests that people in areas with higher prevalence of infectious diseases, adapt collectivistic coping strategies, such as in-group assortative sociality, out-group avoidance, and less dispersal or over shorter distances to manage external environment threats (Fincher and Thornhill, 2008). Thus, the prevalence of infectious diseases was positively correlated with collectivism in the environment.

Humans constantly experience and react to ambient temperature. Temperature is a crucial environmental factor that is associated with individuals’ habitual behavioral patterns. For example, in extreme weather situations, individuals have to spend most of their time indoors. Several models have been proposed to understand aggression and climate differences. A model of CLimate, Aggression, and Self-control in Humans (CLASH) has been proposed to explain differences within and between countries in aggression and violence in terms of differences in climate (Van Lange et al., 2017). The CLASH model proposes that lower temperatures, and especially larger degrees of seasonal variation in climate, call for individuals and groups to adopt a slower life history strategy, a greater focus on the future (vs. present), and a stronger focus on self-control. Such regional temperature induced difference in individual and collective activities may also influence fundamental dimensions of personality in general. Indeed, a recent study using data from 59 Chinese cities (*N* = 5,587) and 12,499 ZIP-code level locations in the United States (*N* = 1,660,638) revealed that individuals who grew up in regions with more clement temperatures scored higher on personality factors related to socialization and stability (agreeableness, conscientiousness, and emotional stability) and personal growth and plasticity (extraversion and openness to experience), compared with individuals who grew up in regions with less clement temperatures (Wei et al., 2017).

Climato-economic explanations of culture propose that inhabitants in poorer resource environment with more demanding winters or summers become more collectivist, because they adopt risk avoidance strategy and place secure into priority, to confront harsh environmental challenges through collectivistic control and seclusion. It has been shown that greater environmental threats and a greater dearth of resources promote cultural tightness (Triandis, 2018). Geographic differences in the strength of collectivist orientations at the provincial level have been explained by the interactive impact of climato-economic hardships within China (Van de Vliert et al., 2013).

In addition to temperature, air pollution may influence human social activities, such as criminal activity and unethical behavior. Analyses of a 9-year panel of 9,360 cities in United States found that air pollution predicted six major categories of crime (Lu et al., 2018). A national survey of a balanced panel of 25,486 individual respondents over the age of 10 in 2010 and 2014 revealed that exposure to air pollution impedes cognitive performance (Zhang et al., 2018). It is thus reasonable to believe that air pollution may impair individuals’ cognitive control and lead to higher levels of aggression. Taken together, parasite stress, and climate-economic theories may both account for cross-cultural/group variation (Van de Vliert and Murray, 2018).

Social influence looks at how individuals’ thoughts, feelings, and behaviors can be influenced by how people behave in the environments in which they live (Rentfrow et al., 2008; Rentfrow and Jokela, 2016). Individuals’ behaviors and attitudes are affected by social norms shaped by the traditions, customs, lifestyles, and common practices in the environments in which they live (Rentfrow, 2010), thereby contributing to geographical differences in psychological phenomena. Future studies may examine how these existing theories account for big-data based findings and explore other causes of geographical variations in psychological phenomena.

## Geographical Differences in Psychological Phenomena

Under the umbrella of geographical psychology, numerous studies have identified uneven geographical distributions in personality, individualism/collectivism, cultural tightness-looseness, subjective well-being, and other psychological phenomena across nations and across regions within nations (Rentfrow and Jokela, 2016). It is thus important to look at these geographical differences and distributions as they are strongly associated with important political, economic, social, and public health indicators.

### Geographical Differences in Personality

As mentioned previously, personality differences exist across nations and states within nations. Allik and McCrae (2004) used the FFM model to analyze personality across 36 nations and found similar personality profiles in geographically adjacent countries. For instance, North America culture of Canada and America, in comparison with Southeast culture of Philadelphia and Indonesia, is higher in extraversion and openness to experience. Later research extended the FFM model to analyze data across 56 countries and found geographical distributions in personality (Schmitt et al., 2007). Findings revealed that Asian and African countries are higher in conscientiousness, South American and European countries are higher in openness to experience, East Asian countries are lower in openness to experience, and African countries are lower in neuroticism as they show lower scores in anxiety and depression.

Besides national differences, recent studies have looked at personality differences across regions within nations (e.g., United States, United Kingdom, and Russia). For example, research across U.S. states found that neuroticism is highest in Northeastern and Southeastern states and lowest in the Midwest and West Coast states; statewide openness to experience is highest in New England, Mid-Atlantic, and Pacific regions and lowest in Great Plains, Midwest, and Southeastern states (Rentfrow et al., 2008; Rentfrow, 2010). Cluster analysis methods have also been used to examine state-level personality differences (Rentfrow et al., 2013). Personality differences across states form a distinctive geographical pattern classified by three psychological regions. Aside from the United States, Rentfrow and his colleagues investigated personality differences across postal districts of the London Metropolitan area. Their findings revealed that openness to experience is highest in Central London and is inversely correlated to the distance between postal districts and the city area (Jokela et al., 2015). Collectively, the abovementioned personality studies have unanimously indicated significant personality differences across various nations and states within nations.

### Geographical Differences in Individualism and Collectivism

Individualism-collectivism is the most widely accepted psychological dimension with regard to cross-cultural differences (Brewer and Chen, 2007). Individualism is concerned with uniqueness of the self while collectivism is concerned with the relationship between the self and others (Xu et al., 2016). From the end of the 1970s, Hofstede began to introduce individualism-collectivism into intercultural studies and proposed an index for it. His comprehensive research has revealed that individualism scores are higher in the United States, the United Kingdom, the Netherlands, and other European and American countries, and lower in Guatemala, Ecuador, Indonesia, and other Latin American and Southeast Asian countries (Hofstede, 2001). Additionally, research on the associations between baby naming practices and country-level individualism scores revealed that the countries in which Europeans have settled scoring higher on individualism, have lower frequencies of using popular names than European countries (Varnum and Kitayama, 2011).

Vandello and Cohen (1999) found that individualism-collectivism differed across states within nations also. They created an index of collectivism to measure U.S. states and revealed that the *Deep South* showed higher collectivism scores, whereas the Great Plains and the Mountain West states showed higher individualism scores. Yamawaki (2012) adapted Vandello and Cohen’s index of collectivism to measure collectivism variations across 47 prefectures in Japan. The results revealed higher collectivism in Northern and Central Japan, and higher individualism in urbanized states. Likewise, Van de Vliert et al. (2013) found that across 15 provinces in China, provinces with lower-income and more demanding climates showed higher collectivism scores while provinces with temperate climates, irrespective of income, showed lower collectivism scores. Hence, geographical differences at both national and regional (or prefectural, provincial) levels have been identified for the individualism-collectivism dimension.

### Geographical Differences in Cultural Tightness-Looseness

Cultural tightness-looseness refers to the strength of external societal constraints and includes two key components: (1) strength of social norms – the clarity and pervasiveness of norms within societies, and (2) strength of sanctioning – tolerance for deviance from norms within societies (Gelfand et al., 2006). Tight nations have been found to display strong social norms and low tolerance for deviant behavior whereas loose nations display weak social norms and high tolerance for deviant behavior (Gelfand et al., 2011). In addition, investigations of cultural tightness-looseness across 33 nations have revealed that countries with higher ecological and historical threats have tighter cultures as they showed stronger social norms and lower tolerance for deviant behavior. Tight nations were observed to have higher population density, higher projected population increases, and fewer natural resources compared to loose nations.

Aside from national differences in cultural tightness-looseness, Harrington and Gelfand (2014) looked at state-level tightness-looseness by developing an index to measure cultural tightness-looseness across 50 U.S. states. Their findings revealed significant state-level differences in cultural tightness-looseness. Mississippi, Alabama, Arkansas, Oklahoma, and Tennessee were identified as the top five tight states, while California, Oregon, Washington, Nevada, Maine, and Massachusetts were identified as the top five loose states.

### Geographical Differences in Subjective Well-Being

Variations in subjective well-being have been found across nations. Research studies have shown higher subjective well-being in Western European nations, especially Sweden and Denmark, and lower subjective well-being in African and former Communist nations (Oishi and Graham, 2010); these findings remain, even after accounting for a range of control variables, such as income, educational background, etc. For example, Diener (2012) compared East Asian nations with Latin American nations and found the latter displayed higher subjective well-being even after controlling for material conditions.

Besides national variations, subjective well-being has also been shown to vary across regions within nations (Lucas et al., 2013). For example, Rentfrow et al. (2009b) found geographical differences in subjective well-being across U.S. states. Mountain and West Coast U.S. states showed the highest subjective well-being, Eastern American U.S. states showed moderate to high subjective well-being, and Midwest and Southern U.S. states showed the lowest subjective well-being. Furthermore, studies using Twitter data measuring happiness across U.S. states and cities have found that happiness differs geographically, with Hawaii being the happiest state and Louisiana the saddest state (Mitchell et al., 2013). Aside from analyses of the United States, recent research revealed significant differences in subjective well-being across postal districts of the London metropolitan area and found subjective well-being to be higher in affluent regions of Southwest London (Jokela et al., 2015). Variations in subjective well-being would lead to different kinds of social and health outcomes. Therefore, geographical analysis of subjective well-being will be beneficial for shaping positive societal and public-health consequences in nations, states, or communities.

## The Links Between Geographic Level Psychological Variables and Social Outcomes

Geographical differences and distributions of psychological phenomena have been expressed at the geographic level and demonstrated to have important political, economic, social, and public-health outcomes (Rentfrow and Jokela, 2016). Currently, geographical differences in personality, individualism-collectivism, and cultural tightness-looseness are strongly associated with the macro-level geographic indicators.

### Geographic Level Correlates of Aggregate Personality Traits

In addition to merely mapping geographical differences in personality, many researchers have looked at how these variations are associated with political, economic, social, and public-health indicators at national or regional levels. Analyses by McCrae et al. (2005) across 51 countries revealed that nations’ levels of extraversion, openness to experience, and agreeableness were positively correlated with egalitarian commitment, per capita gross domestic product, and human development index. Subsequently, McCrae and Terracciano (2008) examined links between personality traits and indexes of cancer, life expectancy, and a series of health-related variables across 51 countries; analyses revealed significant correlations between personality traits at the national level, such as extraversion and conscientiousness, and health-related variables such as cancer mortality and life expectancy. Aggregate extraversion trait is positively related to cancer mortality only for women and life expectancy for both men and women, meanwhile aggregate conscientiousness trait is positively related to life expectancy for both men and women, controlling for gross domestic product per capita.

There have also been robust findings for associations between personality and other indicators at regional levels too. First, a series of studies revealed that regional personality distributions are significantly associated with political election votes (Rentfrow et al., 2009a, 2015; Rentfrow, 2010). For example, Rentfrow et al. (2009a) examined the 1996, 2000, and 2004 United States. Presidential elections and found that states high in openness to experience and low in conscientiousness had higher percentages of votes for Democratic candidates, whereas states low in openness to experience and high in conscientiousness had higher percentages of votes for Republican candidates. Second, regional personality distributions are strongly associated with regional human capitals (Rentfrow et al., 2008, 2015), economic development (Allik et al., 2009; Yang and Lester, 2016), entrepreneurship rates (Obschonka et al., 2013), and other economic indicators. For instance, Obschonka et al. (2013) proposed an entrepreneurial personality profile (featured by high openness, extraversion, and conscientiousness and low agreeableness and neuroticism) and found a positive association between the entrepreneurial personality profile and entrepreneurial activity in 50 U.S. states and the District of Columbia. These findings converged with analyses of 15 United States. Metropolitan Statistical Areas, and were replicated in 14 regions of Germany and 12 regions of the United Kingdom. Subsequently, Obschonka et al. (2016) used large-scale personality datasets and census statistics data from the United States and the United Kingdom to measure whether economic resistance is associated with macro-psychological factors. Their study found that regions that were more emotionally stable and had a higher prevalence of the entrepreneurial personality profile were more resistant to macroeconomic shocks, such as the Great Recession of 2008.

Third, regional personality distributions are strongly correlated with important social indicators, such as trust (Allik et al., 2009), crime rates (Rentfrow et al., 2008, 2015), and cultural diversity (Rentfrow et al., 2008, 2015). Allik et al. (2009) undertook research in Russia and found that regional levels of trust, which is one facet of Agreeableness in NEO-PI-R, are inversely correlated with the distance between the region and the capital. In the United States, Rentfrow et al. (2008) showed that rates of robbery and murder are positively correlated with state-level openness to experience and extraversion, and negatively correlated with state-level agreeableness. These results remained even after controlling for factors such as income and gender. Finally, regional personality differences have been shown to have significant correlations with mortality rates (Rentfrow et al., 2008, 2015), chronic disease rates (Pesta et al., 2012), suicide rates (McCann, 2010), and other public health indicators. For example, Pesta et al. (2012) found that neuroticism has positive associations with variables measured at the state-level such as diabetes, high blood pressure, coronary heart disease, and other chronic diseases. The findings remained robust when controlling for income, education, and crime rate across states.

In summary, national and regional personality differences have been shown to be strongly linked with important political, economic, social, and public health-indicators in macro environments. It is therefore beneficial to incorporate a geographical perspective into personality cross-sectional studies that may allow us to make valid inferences about causality in future research.

### Geographic Level Correlates of Individualism-Collectivism

Several national-level studies have explored the links between individualism-collectivism and various social and health indicators. For example, Mazar and Aggarwal (2011) revealed a positive correlation between a nation’s collectivism and its population’s propensity to initiate bribes (as indexed by the Transparency International’s Bribe Payers Index). In addition, studies have examined links between individualism-collectivism and health indicators. For example, Diener et al. (2003) found that nations higher in individualism have higher subjective well-being scores but also have higher suicide and divorce rates. Subsequent research by Chiao and Blizinsky (2010) revealed that cultural individualism is positively associated with the prevalence of affective disorders such as anxiety and mood disorder. In addition, the short (S) allele frequency of the serotonin transport functional polymorphism (5-HTTLPR) is positively correlated with the prevalence of anxiety and mood disorder. Hence, even though Asian nations have more individuals carrying the S allele of the 5-HTTLPR, the strong collectivistic culture in Asian nations is a strong buffering factor between the S allelic frequency of 5-HTTLPR and the prevalence of affective disorders geographically.

Aside from national differences, individualism-collectivism differences have been examined at the regional level too. For example, Vandello and Cohen’s (1999) research across 50 U.S. states found that state-level collectivism is negatively correlated with alcohol abuse and suicide rate but not correlated with coronary heart disease. Thus, not only does individualism-collectivism identify cultural differences across nations and regions, this dimension can be used for research that aim to improve the well-being of societies and individual health.

### Geographic Level Correlates of Cultural Tightness-Looseness

National differences in cultural tightness-looseness could affect the macro-environment in various ways. For example, Gelfand et al. (2011) found that across 33 nations, tight nations have a higher likelihood of governing in autocratic ways to suppress dissent, have higher control over media institutions, and enforce greater deterrence for group activities than do loose nations. Despite these findings, tight nations have lower murder rate, burglary rate, and overall crime rates in comparison with loose nations. Furthermore, tight nations displayed stronger religious beliefs and belief in the importance of God. Lastly and accordingly, citizens from tight nations had higher self-regulatory strength and higher self-monitoring ability in terms of individually psychological adaptation. Following the work above, Aktas et al. (2016) examined how cultural tightness–looseness influences perceptions of effective leadership across 27 countries. Analyses showed that cultural tightness is negatively related to the endorsement of charismatic leadership and positively associated with the endorsement of autonomous leadership, even controlling for several important societal and organizational level indicators.

For regional-level studies, Harrington and Gelfand (2014) analyzed the association between cultural tightness-looseness with social organization, creativity, equality, and happiness across U.S. states. In terms of social organization, state-level tightness-looseness is negatively correlated with social disorganization. Specifically, loose states were found to have stronger social instability while tight states are associated with stricter law enforcement, more state and local law enforcement full-time employees, and lower homelessness rates. Tight states are associated with lower levels of creativity, as reflected in such measures as fewer fine artists, greater behavioral constraints, and narrower behavioral options. In terms of equality, tight states had stronger discrimination, lower political equality, and lower legal equality but tightness-looseness is not correlated with economic inequality. In terms of psychological health, cultural tightness is negatively correlated with wellbeing, and positively correlated with excessive constraint, and behavioral restriction at the state level of United States (Harrington and Gelfand, 2014). Even when controlling for GDP per capita, state-level cultural tightness is negatively correlated with online happiness expression, based on Twitter data. However, loose states were found to have more illicit drug use, more alcohol binge drinking, and poor financial self-control (Harrington and Gelfand, 2014).

Across the Pacific Ocean, in a more recent work, Chua et al. (2019) mapped cultural tightness and looseness across 31 provinces in China, and revealed its relations to important regional indicators. They found that provincial tightness is positively associated with governmental control, religious practices, and restrictions in daily life. Contrast to prior findings in United States, cultural tightness in China is positively related to economic growth, urbanization, higher life expectancy and more tolerance to the LGBT, and gender equality. Cultural tightness at the provincial level has negatively associated with rates of radical innovations and positively linked to rates of incremental innovations.

Thus, cultural tightness-looseness analyses across geographical regions help to identify many negative consequences at the nation and state levels, which could provide insights as to how to reduce these unwanted macro-level outcomes.

## Implications of Macro-Level Geographical Perspective for Psychological Research

The research reviewed above suggests that geographical perspective can be instructive for understanding human behavior (Rentfrow, 2013). The value of this perspective has been best exemplified in the fields of economic geography, social epidemiology, and political geography, where geographic analyses are already a core part of the fields. For example, economic geography looks at how economic prosperity and job growth are affected by geographically spatial distribution. In social epidemiology, social determinants of health and its incidence are investigated across geographical regions. Similarly, political geography examines how population demographics and historical migration patterns influence election returns and elected officials’ quality of representation. The geographical perspective in psychological research has provided a broader perspective for understanding the interaction between psychological phenomena and their spatial components.

Geographical psychology focuses on the spatial distribution of psychological phenomena at the macro level and their relations to important social outcomes and features of the macro environment. Its perspective overlaps with that of cross-cultural psychology, socio-ecological psychology, and environmental psychology, meanwhile geographical psychology contributes exclusively to psychological science by its own highlights on theorizing and research. Both geographical psychology and cross-cultural psychology concern with associations between psychological phenomena and the broader environment. Cross-cultural psychology focuses more on cultural symbols, norms and practices as the external environment (or situation) that affects human behaviors and thoughts (Oishi and Graham, 2010), rather than socio-econo-political dimensions of socio-ecological environment and their spatial distributions, on which geographical psychology puts much emphasis.

Methodologically, geographical psychology aggregates large-scale questionnaire data, uses cross-sectional design and discovers how psychological phenomena interact with the macro environment. And cross-cultural psychology mainly uses experimental methods to demonstrate how culture influences individual psychological processes. Over the past decade, much attention of cross-cultural psychologists has been drawn to ecological causes of cultural psychological constructs (Sng et al., 2018). Therefore, they began to introduce geographical and socio-ecological perspective to cross-cultural psychology. That’s why much work on geographical or socio-ecological psychology has been conducted by cross-cultural psychologists so far.

In contrast, geographical psychology and socio-ecological psychology all explore how psychological processes are influenced by the macro, objective, concrete ecological environments and social conditions. However, unlike socio-ecological psychology, geographical psychology does pay much attention to how psychological phenomena distribute spatially and their relations to important geographic consequences. In addition, although overlapping a few topics with (early) environmental psychology, geographical psychology extends and go beyond environmental psychology’s lens, which concentrates on the features of the immediate built and natural environments in relations with individual behaviors and thoughts, to devote more attention to geographic clustering of psychological phenomena at different regional levels (e.g., cities, states, and regions) and socio-econo-political dimensions of the broader environment. In contrast, environmental psychology underlines much on the influence of individuals on the natural environment, ways to encourage people’s pro-environmental behavior, and what and how accessible policies could better maintain a sustainable environment, considering the tremendous challenges of global warming and climate change that human mankind encounters currently (Steg and de Groot, 2019).

Geographical psychology is important in explaining social and psychological phenomena at the macro-level. It is highly associated with numerous macro-level indicators such as politics, economics, and public health, which have intriguing findings when analyzed at different geographic levels (Rentfrow et al., 2008). Furthermore, it allows investigating the psychological phenomena that occur with relatively small probability among individuals, and it could zoom out the research view to the broader level. Such as, studying rare mental diseases may present challenges in obtaining enough valid samples and unpacking their antecedents at the individual level, but when observing the geographically distributional incidence of the rare mental diseases at the macro-level perspective, research may have a distinct larger picture to investigate them.

Furthermore, geographical psychology research provides scientific findings with important relevance of policy making. The psychological research findings at the individual level cannot be assumed to be identical at the macro-level and be necessarily implicative for public policy, as it could result in the “individualistic fallacy” or “reverse ecology fallacy,” if there is a lack of evidence at the group level (Inglehart and Welzel, 2003; Rentfrow, 2010). Hence, research at macro-levels tests the applicability of research findings at the individual level. For example, studies have found that obesity-related public-health policy was effective in reducing individual weight but ineffective in reducing obesity rates at the macro-level (Jeffery, 2001). Therefore, psychological research at the geographical level allows better identification of the effectiveness of public health policies.

Although we wouldn’t directly generalize the individual-level research findings to the geographical levels, and vice versa, much work on geographical psychology has been guided and informed by previous individual-level findings to propose theoretical hypotheses and predictions (Rentfrow, 2010). Considering a profounder tradition focusing on micro perspective and plenty of theories and research in the field of psychology, it is not strange that geographical psychology is cultivated more by individual-level psychological research. Nevertheless, the findings of geographical psychology could provide valuable insights to individual psychology too. As mentioned above, for example, Lu et al. (2018) found that air pollution positively predicted the geographical variations of criminal rates across the United States via the analyses of a 9-year panel of 9,360 cities in United States. Thus, they proposed an individual-level prediction that air pollution could increase criminal and unethical behavior. Three further experiments acknowledged the causal association between experimentally perceiving a polluted environment and unethical behavior, and showed that anxiety mediated this relationship.

## Caveats and Future Directions

There is much room for development of geographical analysis in psychology. We uphold that multi-level analysis, identifying causality at macro-level, and incorporating big data techniques deeper are the most promising directions for future research in geographical psychology.

### Geographical Analysis of Psychological Phenomena at Different Levels

The research on geographical psychology summarized above adds to the debate on whether cultural differences can be reduced to individual differences (Na et al., 2010). It is worth noting that the constructs utilized to study group differences at the national and regional levels were originally developed to describe individual differences, such as the big five personality traits. As a consequence, attributes that can differentiate individuals may not be the best ones to capture differences at a group-level. Similarly, national characteristics may not be meaningful individual-difference constructs. Correlations at one level pose no constraints on correlations at another level. Therefore, the group differences revealed in geographical analysis need to be interpreted with caution. Further studies may also develop constructs and measurement scales that are tailored to group-difference research at different levels to link features and dimensions of macro environments (Hofstede and McCrae, 2004).

Future research should also consider conducting analyses across communities, states, regions, and nations, and undertake comparisons of these findings across levels. Multilevel models can be constructed based on the findings so as to search for mechanisms that may explain how environments affect psychological development. For example, Stavrova (2015) used a multilevel regression analysis (individual and state level) to conduct research on neuroticism and life satisfaction across 16 German states. His findings revealed that individual-level life satisfaction is affected by state-level neuroticism even after controlling for individual-level neuroticism. In fact, the geographical perspective could help provide a broader perspective and offer theoretical explanations for the “nature vs. nurture” debate. And the “Culture × Person × Situation” hypothesis could be explored (e.g., Liu et al., 2018). For instance, Using data from a sample of over 10,000 Facebook users across U.S. states, Liu et al. (2018) examined how state-level cultural tightness–looseness interacts with individuals’ social network density on his/her online emotional expression. The analyses showed that individuals in culturally tight states as well as in dense (vs. sparse) networks, are more likely to express positive emotions on Facebook, while it was reversed in culturally loose states. However, there was no such “Culture × Situation” interaction for individuals’ negative emotional expression.

Average differences of individual’s encultured response profile of variables (e.g., values, beliefs, etc.) at different levels, were considered *cultural differences* in cross-cultural psychology, meanwhile, prior research found that variance in cultural variables, such as values, between individuals is much greater than between cultures (Fischer and Schwartz, 2011). On the other hand, these cultural dimensions show fine predictive and discriminant validity (e.g., Schwartz, 2008; Taras et al., 2010). Smith and Bond (2019) proposed *culture* should be re-conceptualized as normative group constraints, and defined the so-called *cultural differences* as “process” variables, through the institutional–normative constraints and affordances socialization into all kinds of cultural groupings at different levels, affecting individual functioning. We agree on that to a large extent and believe the same logic could be introduced to geographical psychology. As such, geographic clustering and averaging aggregations of psychological variables (or phenomena) at different levels, are not just subjects to be analyzed, but also normative group constraints affecting individual thoughts, feelings, and behaviors. Utilizing data from over 500 thousands of residents of 860 cities in United States, Bleidorn et al. (2016) examined whether the fit between individuals’ personality traits and averaging aggregation of personality traits of the city’s where they live, would predict individuals’ self-esteem. The results confirmed the effects of person-city personality fit on self-esteem. Hereby, the prevalent traits of the city’s inhabitants are the normative group constraints affecting individual functioning, i.e., self-esteem.

Thus, multi-levels of geographical analysis not only provide novel findings (Rentfrow, 2010), they may also introduce novel insights to theorizing and research in psychological science.

### Identifying Causality in the Macro-Level Geographic Analysis

Unlike experimental studies that typically control scenarios and identify confounding variables, the researches of geographical psychology, examining the relationship between aggregated variables, usually use cross-sectional design and analysis to literally establish the statistically regressive “causality” at macro levels. But we could still summarize and propose four principles and suggestions to make causal assumptions to some extent.

First, following the consensus or custom of previous studies, scientific regional research has always argued that regional education, income, gender, ethnic diversity and urbanization are important antecedents of regional differences (e.g., Axelrod, 1986; Erikson et al., 1993; Heppen, 2003). Second, important historical and ecological variables, such as the historical prevalence of infectious diseases and the climatic demands (or clement temperatures), could be assumed as antecedents of individuals’ or aggregate psychological phenomena, rather than as consequences. As mentioned in section three of the paper, for instance, Wei et al. (2017) conducted two large-scale studies in China and the United States, and analyses showed that individuals who grew up in regions with more clement temperatures (that is, closer to 22°C) scored higher on personality factors related to personal growth and plasticity (extraversion and openness to experience) and socialization and stability (agreeableness, emotional stability, and conscientiousness).

Third, causality identifying methods in econometrics and quantitative history, such as Granger causality tests and instrumental variables method, could be introduced into geographical psychology. Obschonka et al. (2018) examined relationships between the historical employment share in large-scale coal-based industries and today’s regional variation in personality and well-being. They found that the historical local dominance of large-scale coal-based industries predicts today’s markers of psychological adversity (lower Conscientiousness, higher Neuroticism, and lower life satisfaction and life expectancy). An instrumental variable analysis, using the historical location of coalfields, supported the causal assumption behind these effects. Also, Obschonka et al. (2017) examined the link between strategic bombing of 89 German cities and today’s regional levels in neurotic traits and related mental health problems. They found negative effects of strategic bombing on regional trait depression and mental health problems, controlling for a host of economic factors and social structure.

Last but not least, longitudinal data, which were usually collected and used by developmental psychologists, allow to identify development trajectories and underlying mechanisms of psychological phenomena. Geographical psychology could also try on this kind of data, even though which are very difficult to collect and obtain, and combine them with external geographic data to deepen our understanding of the dynamic relations between psychological phenomena and macro environments.

### Online Survey Platform and Big Data Techniques in Geographical Psychology Research

One obvious limitation of doing research at the large scale level is the high cost of data collection. Geographical psychology research has benefitted greatly from the development of online survey data collection platforms. The platforms could provide cost effective, reliable, and high-quality data (Gosling et al., 2004; Buhrmester et al., 2018), and it has reduced effort and time in measuring psychological constructs across geographic regions. For example, research by Rentfrow et al. (2008, 2015) on geographical personality differences in the United States and the United Kingdom used web-based personality questionnaires platform (the Gosling-Potter Internet Personality Project; for details, see Rentfrow et al., 2008) to collect large samples data across the United States and the United Kingdom, and was proven to have high reliability and validity (Rentfrow et al., 2008; Rentfrow, 2010; Rentfrow and Jokela, 2016).

As mentioned above, big data research in regional differences of psychological phenomena proxied by social media or online query data, has prompted reinvigorating of geographical perspective currently in psychology, to some extent. There is much potential for big data methodology to contribute to geographic psychology. The development of big data techniques, such as machine learning, natural language processing, and sentimental analysis, has allowed researchers to investigate and represent human psychological phenomena or constructs increasingly via human’s online digital traces data (Qiu et al., 2017). For instance, scholars established a method for assessing personality using an open-vocabulary analysis of language from social media Facebook.com. They compiled the written language from over 66,000 Facebook users and their questionnaire-based self-reported Big Five personality traits, and then built a predictive model of personality based on their online language by means of machine learning (Park et al., 2015). More progressively, Wu et al. (2015) further developed the method of assessing personality dimensions using merely a generic digital footprint (Facebook Likes) data, and outperformed humans in personality judgment and predicting some life outcomes. Therefore, future research could consider using large-scale social media users’ digital traces data to acquire individuals’ personality features and then aggregate personality means across geographical regions. Likewise, if social media data can be used to predict and represent the personality characteristics of geographical regions, they could be applied to explore the geographical distributions and even temporal variations of other important psychological constructs. Thus, geographical psychology research should benefit more from the development of big data techniques, to further examine the emerging and evolving mechanisms of geographical differences in psychological phenomena.

## Author Contributions

All authors contributed to the writing of the manuscript and approved the final manuscript to be published. HC and KL wrote the first draft of the manuscript. RY and LH revised the manuscript.

## Conflict of Interest

The authors declare that the research was conducted in the absence of any commercial or financial relationships that could be construed as a potential conflict of interest.
